# The associations between objectively-determined and self-reported urban form characteristics and neighborhood-based walking in adults

**DOI:** 10.1186/1479-5868-11-71

**Published:** 2014-06-04

**Authors:** Elizabeth Jack, Gavin R McCormack

**Affiliations:** 1 Department of Community Health Sciences, Faculty of Medicine, University of Calgary, 3280 Hospital Drive, N.W, Calgary, Alberta T2N 4Z6, Canada; 2 Institute for Public Health, University of Calgary, Alberta, Canada

## Abstract

**Background:**

Self-reported and objectively-determined neighborhood built characteristics are associated with physical activity, yet little is known about their combined influence on walking. This study: 1) compared self-reported measures of the neighborhood built environment between objectively-determined low, medium, and high walkable neighborhoods; 2) estimated the relative associations between self-reported and objectively-determined neighborhood characteristics and walking and; 3) examined the extent to which the objectively-determined built environment moderates the association between self-reported measures of the neighborhood built environment and walking.

**Methods:**

A random cross-section of 1875 Canadian adults completed a telephone-interview and postal questionnaire capturing neighborhood walkability, neighborhood-based walking, socio-demographic characteristics, walking attitudes, and residential self-selection. Walkability of each respondent’s neighborhood was objectively-determined (low [LW], medium [MW], and high walkable [HW]). Covariate-adjusted regression models estimated the associations between weekly participation and duration in transportation and recreational walking and self-reported and objectively-determined walkability.

**Results:**

Compared with objectively-determined LW neighborhoods, respondents in HW neighborhoods positively perceived access to services, street connectivity, pedestrian infrastructure, and utilitarian and recreation destination mix, but negatively perceived motor vehicle traffic and crime related safety. Compared with residents of objectively-determined LW neighborhoods, residents of HW neighborhoods were more likely (p < .05) to participate in (odds ratio [OR] = 3.06), and spend more time, per week (193 min/wk) transportation walking. Perceived access to services, street connectivity, motor vehicle safety, and mix of recreational destinations were also significantly associated with transportation walking. With regard to interactions, HW x utilitarian destination mix was positively associated with participation, HW x physical barriers and MW x pedestrian infrastructure were positively associated with minutes, and HW x safety from crime was negatively associated with minutes, of transportation walking. Neither neighborhood type nor its interactions with perceived measures of walkability were associated with recreational walking, although perceived aesthetics was associated with participation (OR = 1.18, p < .05).

**Conclusions:**

Objectively-determined and self-reported built characteristics are associated with neighborhood-based transportation walking. The objectively-determined built environment might moderate associations between perceptions of walkability and neighborhood-based transportation walking. Interventions that target perceptions in addition to modifications to the neighborhood built environment could result in increases in physical activity among adults.

## Background

Walking can be undertaken by most adults for transportation or leisure, requires no special skills or equipment, and more importantly provides physical and psychological health benefits [1,2]. Neighborhoods are a popular setting where walking is undertaken. Perceived and objectively-determined neighborhood built characteristics such as the proximity and mix of land uses and destinations, street and pedestrian connectivity, residential and population density, personal and traffic safety, and aesthetics are important correlates of walking [3]. However, perceived (self-reported) and objectively-determined measures of the same environmental attributes often show only weak-to-moderate agreement [4-9] and in some cases are found to be differentially associated with physical activity outcomes [10-13]. Therefore, perceived and actual characteristics of the built environment both need to be considered when investigating the role of neighborhood environments in influencing physical activity.

Perceptions of the built environment are likely to be influenced by the exposure to, or experience within, the “actual” built environment. Individual-level factors such as attitudes, beliefs, self-efficacy, preferences, habit, past experiences, health, and socioeconomic status can also influence perceptions [14,15]. Several studies have found that physical activity-related cognitions such as perceived behavioral control, intention, and attitude mediate associations between the built environment and physical activity [16-20]. This evidence suggests that perceptions of the built environment, also a cognition, might *mediate* built environment-physical activity causal pathways. Indeed, perceptions of the built environment have been postulated to mediate the association between the objectively-determined built environment and physical activity [7,21]. Associations between perceptions of the built environment and physical activity while controlling for the objectively-determined built characteristics have also been found [8,22,23]. 

Few studies however, have examined whether perceptions of the built environment mediate the relationship between objectively-determined built environmental characteristics and physical activity. Van Dyke et al. [24] found that among socioeconomically disadvantaged women, perceptions of aesthetics and social cohesion mediated the association between the objectively-determined neighborhood characteristics (i.e., a street connectivity and destination density score) and leisure-time walking. In the same study, neighborhood perceptions mediated associations between the objectively-determined built characteristics and transportation walking [24]. Moreover, longitudinal evidence from a natural experiment suggest that changes in neighborhood-based recreational walking associated with changes in the built environment (mix of local recreational destinations) might be mediated by perceived neighborhood attractiveness [25].

Perceptions of the built environment might also *moderate* the effect of the objectively-determined built environment on physical activity and vis-a-versa. Indirect evidence of moderation is provided by several studies finding interactions between the built environment and other physical activity-related cognitions and physical activity [26,27]. Higher access to recreational facilities and parks, and more favourable aesthetics have been found to compensate for the lack of individual disposition (i.e., self-efficacy, social support, enjoyment, perceived benefits and barriers) in determining physical activity [26]. Interactions between the perceived and objectively-determined built environment might also influence physical activity, yet the few studies that have investigated this relationship provide mixed evidence. For instance, Gebel et al. [6] found that levels of walking was higher among residents of objectively-determined low walkable neighborhoods who perceived the walkability of their neighborhood to be high. Elsewhere, the objectively-determined distance from respondent’s homes to the coast did not moderate the relationship between longitudinal changes in neighborhood perceptions and neighborhood-based walking [28]. Michael and Carlson [29] found that objectively-determined walkability did not modify the effects of a neighborhood-based walking intervention, but they did find increases in walking as well as increases in perceptions of neighborhood problems in the intervention group.

A better understanding of how the objectively-determined and perceived built environment together influence walking could inform interventions designed to increase physical activity in low and high walkable neighborhoods [30]. The objectives of this study were to: 1) compare self-reported measures of the neighborhood built environment between objectively-determined low, medium, and high walkable neighborhoods; 2) estimate the relative associations between self-reported and objectively-determined neighborhood characteristics and walking and; 3) examine the extent to which the objectively-determined built environment moderates the association between self-reported measures of the neighborhood built environment and walking.

## Method

### Sampling and study design

The study methodology has been fully described elsewhere [19,31]. Briefly, a random cross-section of Calgary (Alberta, Canada) residents (≥18 years of age) participated in telephone-interviews during either August-October 2007 (n = 2199, response rate = 33.6%) or January-April 2008 (n = 2223, response rate = 36.7%). Telephone-interviews captured information about neighborhood-based physical activity behavior, psychosocial and sociodemographic characteristics, reasons for moving to the current neighborhood (i.e., neighborhood self-selection), and neighborhood tenure. N = 1967 participants who completed the telephone-interviews also completed and returned a follow-up postal survey. The postal survey captured information about perceptions of neighborhood walkability, physical activity behaviors, and additional socio-demographic characteristics. The University of Calgary Conjoint Health Research Ethics Board approved this study.

### Neighborhood-based walking

Walking items in the postal survey were adapted from the International Physical Activity Questionnaire (IPAQ). The IPAQ items provide a reliable measure of physical activity undertaken in the past 7-days [32]. For this study, IPAQ items were modified to capture minutes of “neighborhood-based” (i.e., everywhere within a 15-minute walk of home) transportation and recreational walking. Respondents who reported walking <10-minutes/wk were coded as “non-walkers” and those reporting ≥10 minutes/wk were coded as “walkers”. Data cleaning recommendations for the IPAQ suggest that values less than 10 minutes of activity should be excluded from summary estimates (http://www.ipaq.ki.se/scoring.pdf).

### Socio-demographic characteristics, walking attitude, and residential self-selection

Socio-demographic information captured included: gender, age, home ownership status (home owner or renter), highest level of education completed (less than high school, high school, college/technical school, undergraduate, or graduate), number of children <18 years of age, and time (in years) spent living in the neighborhood. Attitude towards walking was a composite variable based on the average response across six items. Items captured level of participant agreement (i.e., strongly disagree, disagree, agree, or strongly agree) about whether walking regularly in the near future would be foolish, beneficial, useful, enjoyable, relaxing and interesting (test-rest reliability r = 0.53-0.74 and internal consistency Cronbach’s alpha(α) = 0.83). Nineteen items captured the importance (not at all important, somewhat important, or very important) of neighborhood characteristics in the respondents’ decision to locate to their current neighborhood. Items were aggregated into four scales based on results from principal component analysis. A four factor solution provided the best fit to the data and included the following scales: 1) *access to places that support physical activity* (n = 6 items; variance explained (VE) = 29.7%; α = 0.79); 2) *access to local services* (n = 4 items; VE = 9.8%; α = 0.61); 3) *sense of community* (n = 4 items; VE = 8.5%; α = 0.71), and 4) *ease of driving* (n = 2 items; VE = 6.5%; α = 0.54). Three items were excluded from further analysis due to cross-loading onto multiple scales. The neighborhood self-selection and attitude scales were transformed into z-scores.

### Self-reported neighborhood walkability

Perceptions of neighborhood walkability were captured using the Abbreviated Neighborhood Walkability Scale (NEWS-A) [33]. For consistency with the walking items, ‘neighborhood’ was defined as anywhere within a 15-minute walk from home. Using a principal component analysis (with varimax rotation), a seven factor solution best fit the self-reported walkability data: *safety from crime* (α = 0.71); *neighborhood aesthetics* (α = 0.77); *access to services* (α = 0.65*); street connectivity* (α = 0.48); *pedestrian infrastructure* (α = 0.33); *motor vehicle traffic safety* (α = 0.55), and; *physical barriers* (α = 0.35) [see 19]. Two additional indices, recreation destination and utilitarian destination mix within 15-minutes of home were also created. *Recreation destination mix* includes the self-reported count of destinations (gymnasium, recreation centre, park, and trail) that likely support physical activity for recreation while *utilitarian destination mix* includes the self-reported count of destinations (book store, clothing store, cafe, bank, transit, drug store, post office, supermarket, convenience store, fast food restaurant, hair salon/barber, video store, library, dry cleaner, farmers market, school, and hardware store) that might support transportation walking. Positive associations between self-reported and objectively-measured mix of destinations and walking have been reported elsewhere [3,34]. All neighborhood walkability variables were standardized and we made the assumption that higher scores reflected more positive perceptions with regard to the supportiveness of the neighborhood environment for walking.

### Objectively-determined neighborhood walkability

Three neighborhood types (low walkable (LW), medium walkable (MW), and high walkable (HW)) with homogeneous built characteristics were identified using cluster-analysis [31]. Briefly, respondent’s postal codes were geocoded and used as proxy locations for their household address. Canadian six digit postal codes typically include 15–20 households and are reasonably accurate with regard to being used as a proxy for residential household location [35]. Built characteristics included: walkshed area (in km^2^), number of businesses/km^2^, transit stops/km^2^, mix of park types/km^2^, mix of recreational facilities/km^2^, sidewalk length in meters/km^2^, and total population/km^2^ within 1.6 km of the respondent’s household (via the street network) and percentage of green space and path/cycleway in meters/km^2^ within the neighborhood administrative boundary. LW neighborhoods had a relatively low count of destinations, low street connectivity, low population density, low transit stop access, low mix of park types, low sidewalk availability, high green space, high path/cycleway availability and low recreational destination mix. MW neighborhoods also tended to have a relatively low count of destinations, low population density and low transit access; however they also had medium connectivity, high park mix, high sidewalk availability, medium green space, low path availability and high recreational destination mix. Finally, HW neighborhoods were found to have a high number of destinations, high connectivity, high population density, high transit access, high park mix, high sidewalk availability, low green space, high path availability and high recreational destination mix [31].

### Statistical analyses

One-way Analysis of Variance (ANOVA) for continuous variables and Pearson’s Chi-square for categorical variables were used to compare respondent socio-demographic characteristics, walking, and self-reported walkability between the three objectively-determined neighborhood types. Bonferroni-adjusted pairwise comparisons were undertaken for statistically significant (p < .05) ANOVA or Pearson’s Chi-square tests. Where the assumption of equality of group variances was violated and a statistically significant ANOVA found, a Kruskal-Wallis One-way ANOVA (non-parametric test) was undertaken to confirm the result.

Multivariate binary logistic regression (odds ratios [OR] and 95% confidence intervals [CI]) was used to regress neighborhood-based walking participation (i.e., ‘non-walker’ or <10-minutes versus ‘walker’ ≥ 10-minutes in the past seven days) on neighborhood type (i.e., LW, MW and HW) and the nine self-reported walkability variables, adjusting for the covariates (i.e., socio-demographic characteristics, attitude towards walking, and the four neighborhood self-selection variables). For those who reported participating in neighborhood-based walking in the past 7-days (i.e., ‘walkers’), Generalized Linear Models (GZLM with gamma distribution and identity link function) were used to regress minutes of neighborhood-based walking on objectively-determined neighborhood type and self-reported walkability variables, adjusting for the covariates. To aid in the interpretation, GZLM estimates for neighborhood-type were reported as marginal means (with 95% CIs), while β-coefficients (i.e., slope parameters with 95% CIs) were reported for the self-reported walkability variables. For models predicting participation in, and duration of, walking, blocks of variables were entered into the models in sequence beginning with objectively-determined neighborhood type then the self-reported walkability variables. The sequential entry of variables in the model allowed attenuation in the association between objectively-determined neighborhood type and walking after adjustment for self-reported walkability variables to be observed. Interaction terms between neighborhood type (i.e., LW, MW and HW) and the nine self-reported walkability variables were also created. The interaction terms were entered in the fully-adjusted main effects models with backwards stepwise removal of statistically non-significant interaction terms (p < .10).

## Results

### Sample characteristics

The analysis included n = 1875 cases with complete telephone and postal survey data. The majority of respondents resided in LW (56.8%), followed by MW (36.1%) and HW (7.1%) neighborhoods (Table 1). MW neighborhoods included fewer 18–39 year olds and more ≥60 year olds, compared with LW and HW neighborhoods (p < .05). LW neighborhoods included higher proportion of homeowners and respondents with dependents <18 years of age compared with MW and HW neighborhoods (p < .05). Mean (±standard deviation) tenure was 17.1 ± 14.2 years in MW, 10.7 ± 9.9 years in LW, and 9.2 ± 9.2 years in HW neighborhoods (p < .05). No statistically significant differences were found in *attitude towards walking* between the three neighborhood types (Table 1). Neighborhood characteristics associated with sense of community and easy of driving were significantly (p < .05) less important in neighborhood selection among those residing in HW versus MW and LW neighborhoods (Table 2). However, neighborhood characteristics associated with *access to services* were more important for choosing to reside in a HW versus a LW neighborhood (2.33 ± 0.54 vs. 2.06 ± 0.50, p < .05).

**Table 1:** Socio-demographic characteristics, attitude and walking behavior by high walkable (HW), medium walkable (MW), and low walkable (LW) neighborhood types

	**Total Sample**	**HW**	**MW**	**LW**
	**(n = 1875)**	**(n = 134)**	**(n = 676)**	**(n = 1065)**
**Socio-demographic characteristics**				
Gender (% women)	62.2	56.7	60.9	63.7
Age in years (%)				
18 to 39^a^	25.9	28.4	20.6	28.9
40 to 59	52.8	50.0	44.1	44.7
≥60^a,b^	29.3	21.6	35.4	26.4
Highest education completed (%)				
Less than high school^a^	4.9	3.7	7.4	3.4
High school	24.9	25.4	25.4	24.5
College/technical school	25.5	20.9	25.1	26.3
University - Undergraduate^a,b^	29.8	36.6	25.3	31.7
University - Postgraduate	15.0	13.4	16.7	14.1
Home owner (%)^a,b,c^	86.3	57.5	83.4	91.8
Dependents (% ≥1 dependents)^a,b,c^	34.1	18.7	28.8	39.4
Years lived in neighborhood (mean ± SD)^a,b^	12.87 ± 12.02	9.20 ± 9.18	17.08 ± 14.22	10.67 ± 9.91
Attitude towards walking (1.0-5.0 scale; mean ± SD)	4.33 ± 0.55	4.36 ± 0.58	4.30 ± 0.54	4.35 ± 0.54
**Neighborhood-based walking**				
No walking for transportation (% 0 times in last 7 days)^a,b,c^	59.8	33.6	55.6	65.7
Minutes of walking for transportation (mean ± SD)^b,c^	106.6 ± 117.4	177.8 ± 166.2	102.5 ± 104.5	92.6 ± 106.8
No walking for recreation (% 0 times in last 7 days)	43.2	56.3	43.8	42.4
Minutes of walking for recreation (mean ± SD)	166.7 ± 159.7	175.3 ± 206.4	164.0 ± 158.5	167.3 ± 154.4

^a^ = LW significantly differs from MW (p < .05) based on One Way ANOVA (continuous variables) or Pearson’s chi-square (categorical variables) with Bonferroni–adjusted pairwise comparison.

^b^ = MW significantly differs from HW (p < .05) based on One Way ANOVA (continuous variables) or Pearson’s chi-square (categorical variables) with Bonferroni–adjusted pairwise comparison.

^c^ = LW significantly differs from HW (p < .05) based on One Way ANOVA (continuous variables) or Pearson’s chi-square (categorical variables) with Bonferroni–adjusted pairwise comparison.

**Table 2:** Mean scores for self-reported neighborhood walkability and reasons for moving to the current neighborhood between high walkable (HW), medium walkable (MW), and low walkable (LW) neighborhood types

	**Neighborhood type**		
	**HW (n = 134)**	**MW (n = 676)**	**LW (n = 1065)**
	**mean ± SD (median)**	**mean ± SD (median)**	**mean ± SD (median)**
**Self-reported neighborhood walkability (1.0-4.0 scale)**			
Access to services^a,c^	3.47 ± 0.63 (3.67)	3.35 ± 0.68 (3.67)	3.09 ± 0.73 (3.21)
Physical barriers^a^	3.51 ± 0.63 (4.00)	3.49 ± 0.63 (3.50)	3.40 ± 0.65 (3.50)
Street connectivity^a,b,c^	3.21 ± 0.68 (3.33)	3.07 ± 0.62 (3.00)	2.67 ± 0.62 (2.67)
Pedestrian infrastructure^a,c^	3.09 ± 0.47 (3.00)	3.08 ± 0.46 (3.00)	2.93 ± 0.54 (3.00)
Neighborhood aesthetics^a,b^	2.91 ± 0.66 (3.00)	3.11 ± 0.62 (3.25)	3.00 ± 0.64 (3.00)
Motor vehicle traffic safety^b,c^	2.55 ± 0.61 (2.67)	2.79 ± 0.60 (3.00)	2.81 ± 0.62 (3.00)
Safety from crime^a,b,c^	2.79 ± 0.72 (2.75)	3.24 ± 0.58 (3.25)	3.39 ± 0.51 (3.50)
**Self-reported neighborhood destinations (count of types)**			
Utilitarian destination mix^a,b,c^	10.78 ± 4.96 (12.00)	9.48 ± 4.47 (10.00)	6.97 ± 4.82 (6.00)
Recreation destination mix^a,c^	4.61 ± 2.00 (4.50)	4.60 ± 1.83 (5.00)	3.84 ± 1.66 (4.00)
**Reasons for neighborhood choice (1.0-3.0 scale)**			
Access to places that support physical activity^a^	2.00 ± 0.48 (2.00)	1.99 ± 0.49 (2.00)	2.07 ± 0.51 (2.00)
Access to services^a,c^	2.33 ± 0.54 (2.50)	2.25 ± 0.50 (2.25)	2.06 ± 0.50 (2.00)
Sense of community^a,b,c^	2.10 ± 0.53 (2.25)	2.32 ± 0.48 (2.50)	2.39 ± 0.48 (2.50)
Ease of driving^a,b,c^	1.69 ± 0.63 (1.50)	2.04 ± 0.58 (2.00)	2.11 ± 0.56 (2.00)

^a^ = LW significantly differs from MW (p < .05) based on One Way ANOVA (with Bonferroni-adjusted pairwise comparison).

^b^ = MW significantly differs from HW (p < .05) based on One Way ANOVA (with Bonferroni-adjusted pairwise comparison).

^c^ = LW significantly differs from HW (p < .05) based on One Way ANOVA (with Bonferroni-adjusted pairwise comparison).

### Differences in measures of perceived walkability between objectively-determined neighborhood types

Compared with LW neighborhoods, respondents in HW neighborhoods had significantly (p < .05) higher perceived *access to services* (HW = 3.47 ± 0.63 vs. LW = 3.09 ± 0.73), *street connectivity* (HW = 3.21 ± 0.68 vs. LW = 2.67 ± 0.62), *pedestrian infrastructure* (HW = 3.09 ± 0.47 vs. LW = 2.93 ± 0.54), *utilitarian destination mix* (HW = 10.78 ± 4.96 vs. LW = 6.97 ± 4.82) and *recreation destination mix* (HW = 4.61 ± 2.00 vs. LW = 3.84 ± 1.66), and lower perceived safety from *traffic* (HW = 2.55 ± 0.61 vs. LW = 2.81 ± 0.62) and *crime* (HW = 2.79 ± 0.72 vs. LW = 3.39 ± 0.51) (Table 2). Perceived *access to services*, *pedestrian infrastructure*, and *recreation destination mix* did not significantly differ between respondents residing in HW and MW neighborhoods. LW and MW neighborhoods significantly (p < .05) differed on all perceived walkability variables except for *traffic safety*. Perceived *neighborhood aesthetics* was higher (p < .05) in MW than LW and HW neighborhoods (Table 2).

### Environmental correlates of neighborhood-based transportation walking

Increases in neighborhood walkability were associated with increases in weekly participation and minutes (among those reporting participation) of neighborhood-based transportation walking (HW = 66.4%; MW = 44.4%; LW = 34.3%; p < .05; Table 1). After adjusting for socio-demographic characteristics, attitude towards walking, and reasons for neighborhood selection, respondents in HW and MW neighborhoods were more likely to walk for transportation than those from LW neighborhoods (OR 2.08; 95% CI 1.35, 3.19 and OR 1.40; 95% CI 1.12, 1.75, respectively) (Model 1a; Table 3). The addition of the perceived walkability variables to the model attenuated the associations between MW and HW neighborhoods and neighborhood-based transportation walking (Model 2a; Table 3). In the same model, *access to services* (OR 1.17; 95% CI 1.05, 1.32), *street connectivity* (OR 1.16; 95% CI 1.03, 1.30), and *utilitarian destination mix* (OR 1.25; 95% CI 1.08, 1.44) were also associate with participation in transportation walking. One statistically significant (p < .05) interaction term was found – an increase in perceived *utilitarian destination mix* was associated with an increase in the odds of participating in transportation walking in HW neighborhoods (Model 3a; OR 2.82; 95% CI 1.58, 5.06).

**Table 3:** Logistic regression and Generalized Linear Model estimates for the association between neighborhood type, self-reported walkability and participation in and minutes of neighborhood-based transportation walking in the last 7-days

	**Participation in walking for transportation**			**Minutes of walking for transportation**		
	**(n = 1875)**			**(among those walking ≥1 times/week; n = 754)**		
	**Model 1a**	**Model 2a**	**Model 3a**	**Model 1b**	**Model 2b**	**Model 3b**
	**OR (95% CI)**	**OR (95% CI)**	**OR (95% CI)**	Estimate (95% CI)*	Estimate (95% CI)*	Estimate (95% CI)*
**Objective neighborhood type**						
Low walkable (LW)	Ref.	Ref.	Ref.	114.24 (97.52, 130.96)^c^	119.83 (101.85, 137.82)^c^	117.95 (100.49, 135.40)
Medium walkable (MW)	1.40 (1.12, 1.75)^†^	1.04 (0.82, 1.32)	1.07 (0.83, 1.36)	113.97 (96.96, 130.99)^b^	115.77 (98.59, 132.94)^b^	115.79 (98.77, 132.80)
High walkable (HW)	2.08 (1.35, 3.19)^†^	1.50 (0.94, 2.41)	1.23 (0.71, 2.15)	178.04 (143.66, 212.42)^b,c^	167.36 (131.56, 203.16)^b,c^	140.14 (110.28, 169.99)
**Self-reported neighborhood characteristics**						
Access to services		1.17 (1.05, 1.32)^†^	1.18 (1.05, 1.32)^†^		-1.01 (-7.03, 5.01)	-1.43 (-7.26, 4.40)
Physical barriers		0.93 (0.84, 1.04)	0.93 (0.83, 1.04)		-5.03 (-14.42, 4.37)	-1.11 (-7.59, 5.37)
Street connectivity		1.16 (1.03, 1.30)^†^	1.14 (1.02, 1.29)^†^		-1.28 (-8.22, 5.66)	0.58 (-6.48, 7.63)
Pedestrian infrastructure		1.10 (0.99, 1.23)	1.10 (0.99, 1.23)		-2.37 (-11.49, 6.75)	4.96 (-2.08, 12.00)
Neighborhood aesthetics		1.01 (0.90, 1.13)	1.01 (0.90, 1.14)		5.07 (-1.19, 11.33)	5.52 (-1.27, 12.32)
Motor vehicle traffic safety		1.00 (0.90, 1.12)	1.00 (0.90, 1,12)		7.71 (1.61, 13.82)^†^	6.01 (-0.35, 12.36)
Safety from crime		0.95 (0.84, 1.07)	0.97 (0.86, 1.09)		-8.15 (-19.07, 2.77)	-11.20 (-19.69, -2.72)^†^
Utilitarian destination mix		1.25 (1.08, 1.44)^†^	1.16 (0.98, 1.37)		6.44 (-1.37, 14.26)	5.54 (-2.42, 13.51)
Recreation destination mix		1.15 (1.00, 1.32)	1.13 (0.98, 1.30)		-9.31 (-17.24, -1.38)^†^	-9.70 (-17.64, -1.76)^†^
**Interactions**						
HW x utilitarian destination mix			2.82 (1.58, 5.06)^†^			
HW x physical barriers						42.14 (17.18, 67.09)^†^
MW x pedestrian infrastructure						15.67 (3.83, 27.51)^†^
HW x safety from crime						-32.36 (-54.48, -10.23)^†^

**Model 1a and 1b:** adjusted for age, gender, education, home ownership, dependents, years lived in neighborhood, attitude towards walking and reasons for neighborhood choice (access to places supporting physical activity, access to services, sense of community and ease of driving).

**Model 2a and 2b:** adjusted for model 1 and *self-reported neighborhood walkability*.

**Model 3a and 3b:** adjusted for model 2 with statistically significant interaction terms retained in the model.

* = Estimated marginal means are reported for objective *neighborhood type*; regression coefficients (β) are reported for all other variables.

^**b**^ = MW significantly differs from HW (p < .05) based on marginal mean estimate from GZLM (with Bonferroni-adjusted pairwise comparison).

^**c**^ = LW significantly differs from HW (p < .05) based on marginal mean estimate from GZLM (with Bonferroni-adjusted pairwise comparison).

**†** = p < .05.

After adjusting for socio-demographic characteristics, attitude towards walking, and neighborhood self-selection, weekly minutes of neighborhood-based transportation was higher among residents of high walkable (HW: marginal mean = 178.04 min/week; 95% CI 143.66, 212.42) versus less walkable neighborhoods (MW: marginal mean = 113.97 min/week; 95% CI 96.96, 130.99, and; LW: marginal mean = 114.24; 95% CI 97.52, 130.96) (Model 1b; Table 3). The inclusion of perceived walkability variables in model 2b resulted in a slight attenuation of transportation walking in HW neighborhoods (marginal mean from 178.04 to 167.36 min/week). Furthermore, perceived neighborhood *safety from crime* (β -11.20 min/week; 95% CI -19.69, -2.72) and *recreation destination mix* (β -9.70 min/week; 95% CI = -17.64, -1.76; p < .05) were negatively associated with transportation walking minutes. With regard to interaction effects, in HW neighborhoods only, minutes of neighborhood-based transportation walking was associated with perceived *physical barriers* (β 42.14 min/wk; 95% CI 17.18, 67.09) and *safety from crime* (β -32.36 min/wk; 95% CI -54.48, -10.23). In MW neighborhoods only, minutes of transportation walking was positively associated with perceived *pedestrian infrastructure* (β 15.67 min/wk; 95% CI 3.83, 27.51) (Model 3b; Table 3).

### Environmental correlates of neighborhood-based recreational walking

Weekly participation and minutes spent walking for recreation were not significantly different between the three neighborhood types (Table 4). Nevertheless, after adjustment for all characteristics, perceived *neighborhood aesthetics* (Model 2a: OR 1.18; 95% CI 1.06, 1.32) was positively associated with participation in neighborhood-based recreational walking while perceived *access to services* was negatively associated with minutes of neighborhood-based recreational walking (β -9.13 min/week; 95% CI -17.90, -0.36) (Model 2b). No statistically significant interactions between neighborhood type and self-reported walkability variables were found for recreational walking participation or minutes.

**Table 4:** Logistic regression and Generalized Linear Model estimates for the association between neighborhood type, self-reported walkability and participation in and minutes of neighborhood-based recreational walking in the last 7-days

	**Participation in walking for recreation**		**Minutes of walking for recreation**	
	**(n = 1875)**		**(among those walking ≥1 times/week; n = 1065)**	
	**Model 1a**	**Model 2a**	**Model 1b**	**Model 2b**
	**OR (95% CI)**	**OR (95% CI)**	Estimate (95% CI)*	Estimate (95% CI)*
**Objective neighborhood type**				
Low walkable (LW)	Ref.	Ref.	158.55 (142.61, 174.50)	161.68 (145.07, 178.29)
Medium walkable (MW)	0.96 (0.77, 1.20)	0.90 (0.71, 1.13)	155.55 (138.03, 173.06)	157.31 (139.50, 175.11)
High walkable (HW)	0.88 (0.58, 1.32)	0.82 (0.54, 1.26)	149.11 (116.36, 181.87)	153.04 (118.19, 187.89)
**Self-reported neighborhood characteristics**				
Access to services		0.99 (0.89, 1.10)		-9.13 (-17.90, -0.36)^†^
Physical barriers		1.03 (0.93, 1.14)		6.45 (-2.03, 14.93)
Street connectivity		1.00 (0.90, 1.12)		0.39 (-8.42, 9.19)
Pedestrian infrastructure		0.94 (0.85, 1.04)		-2.34 (-10.54, 5.86)
Neighborhood aesthetics		1.18 (1.06, 1.32)^†^		2.57 (-6.09, 11.24)
Motor vehicle traffic safety		0.96 (0.86, 1.07)		-5.32 (-14.28, 3.65)
Safety from crime		0.96 (0.86, 1.08)		2.59 (-6.06, 11.24)
Recreational destination mix		1.09 (0.98, 1.21)		4.77 (-3.02, 12.56)

**Model 1a and 1b:** adjusted for age, gender, education, home ownership, dependents, years lived in neighborhood, attitude towards walking and reasons for neighborhood choice (access to places supporting physical activity, access to services, sense of community and ease of driving).

**Model 2a and 2b:** adjusted for model 1 and *self-reported neighborhood walkability*.

No interaction terms were statistically significant.

**†** = p < .05.

* = estimated marginal means are reported for objective *neighborhood type*; regression coefficients (β) are reported for all other variables.

## Discussion

Despite previous evidence suggesting a lack of correspondence between self-reported and objective assessments of the built environment [4-9] we found that perceived neighborhood characteristics for the most part were higher (more positive) in objectively-determined high walkable neighborhoods than in less walkable neighborhoods. Furthermore, similar to evidence elsewhere [3] perceived and objectively-determined neighborhood walkability appears to be more important for neighborhood-based transportation versus recreational walking. Specifically, self-reported and objectively-measured characteristics of the neighborhood were independently associated with both weekly participation and minutes of neighborhood-based transportation walking, adjusting for neighborhood self-selection and socio-demographic characteristics. Noteworthy was that we found dence of effect modification, with stronger associations between perceived physical barriers and safety from crime and local transportation walking in HW neighborhoods only and between pedestrian infrastructure and local transportation walking in MW neighborhoods only. In support of findings elsewhere suggesting that the perceptions of built environment mediate the relationship between the objectively-measuredbuilt environment and physical activity [24,25] we found that perceived neighborhood characteristics attenuated the association between the objectively-determined neighborhood type and participation, but not minutes, in weekly neighborhood-based transportation walking.

For most comparisons, differences in perceptions of walkability were found between the three objectively-determined neighborhood types in the expected direction. Compared with respondents from LW neighborhoods, those from HW neighborhoods in general reported their neighborhoods to be more walkable. HW neighborhoods in our study included, among other attributes, high levels of street connectivity and count of businesses [31] which was congruent with respondent’s positive evaluations of street connectivity, access to services and utilitarian destination mix. HW and MW neighborhoods were similar with regard to sidewalk length, the mix of park types and recreational facilities corresponding with the more positive perceptions of perceived pedestrian infrastructure and recreation destination mix within these neighborhood types. Previous studies that have measured concordance between perceived and objectively-measured walkability have found poor to fair agreement [4-7,9], although Arvidsson [8] found higher agreement when overall indices of perceived and objectively-measured walkability were compared. Gebel et al. [6] found that approximately a third of participants living in HW neighborhoods (based on dwelling density, street connectivity, land use mix and retail density) misperceived their neighborhoods to be low walkable. We found that perceived aesthetics and safety (from crime and traffic) were more negative in objectively-determined high walkable versus low walkable neighborhoods. This result is similar to Van Dyke et al. [24] who found that socioeconomically disadvantaged women residing in neighborhoods with high connectivity and destination density perceived their neighborhoods to have poor aesthetics, to have lower social cohesion, and to be less safe compared with women in neighborhoods that had lower connectivity and destination density. In both studies, self-report measures of aesthetics and safety only were captured and were not objectively-determined. Thus perceptions of aesthetics and safety could reflect real differences across objectively-measured neighborhood types or could reflect differences in the way the perceived and objective built environment measures are operationalized. Comprehensive objectively-determined walkability indices that better reflect the range of built characteristics important for supporting and encouraging neighborhood walking are needed.

Different built environmental factors were found to be associated with walking participation (some or none) and duration (minutes). For example, neighborhood aesthetics had a significant positive association with participation in walking for recreation but not with duration among respondents who reported some walking for recreation, independent of objectively-determined neighborhood characteristics. The differences in potential determinants of transportation versus recreational walking, and of initiating walking versus extending the duration of walking among those who already walk, suggest that there are different decision-making processes involved in these activities. These differences have implications for designing future interventions to promote walking for different purposes. Also notable was that self-reported aesthetics only was associated with neighborhood-based recreational walking while objectively-determined neighborhood type did not appear to be associated with recreational walking. The influence of the built environment on walking behavior may be weaker for recreational than for transportation walking [3]. Speculatively, our cross-sectional findings suggest that the increases in recreational walking might be more likely if interventions target individual-level (motivations and perceptions) factors, while increases in transportation walking might be more likely in interventions focus on changing individual-level characteristics in addition to improving neighborhood walkability.

Statistically significant interaction effects in MW or HW neighborhoods but not for LW neighborhoods were found. For those who actually reside in LW neighborhoods, their perceptions of walkability appear to have limited influence on walking behavior compared with resident of MW and HW neighborhoods. Lacky and Kacynski [12] found that while neither perceived or objective proximity to parks alone was associated with participation in park-based physical activity, their interaction was positively associated with physical activity. Giles-Corti et al. [25] found that among those relocating to new neighborhoods, increases in perceptions of neighborhood walkability were positively associated with time spent walking for transportation and recreation, even after adjusting for objectively-determined neighborhood built characteristics. Among other significant interaction effects, we found that higher positive evaluations of physical barriers (higher scale scores represented fewer barriers) was positively associated with transportation walking in high walkable neighborhoods. Improving perceptions about the physical barriers in high walkable neighborhoods, through micro-scale modifications (removal of major pedestrian barriers, reducing steep hills and gradients along walking routes, etc.) might encourage additional amounts of walking among local residents. More research is needed to better understand how specific types of perceived and objectively-determined neighborhood built characteristics might combine to influence neighborhood-based walking as well as other physical activities. Longitudinal studies that attempt to improve perceptions of the built environment among residents living in neighborhoods of differing levels of walkability could provide insight into the relative contributions of perceived versus built neighborhood characteristics on walking.

Counter-intuitively, an increase in self-reported safety from crime was significantly associated with decreased minutes of walking for transportation in HW neighborhoods among those residents who reported some walking. This interaction appears to be inconsistent with evidence suggesting a positive or no relationship between perceived safety and neighborhood-based physical activity [36], but consistent with Michael and Carlon [29] who found that among older adults increases in walking was accompanied by a reduction in positive perceptions of neighborhood problems (incivilities, violent crime, abandoned buildings etc.). One explanation for this finding is that people who are more active in their neighborhood also tend to be more aware of its characteristics due to greater exposure and familiarity. Alternatively, HW neighborhoods tend to have higher population density, and may afford more potential opportunities for undesirable interactions between people. Speculatively, it is also possible that the nature of crimes differ according to neighborhood type, and that the severity of crimes within high walkable neighborhoods might result in perceived personal safety having a stronger influence on resident’s walking decisions. Improving perceptions of safety in high walkable neighborhoods – i.e., through crime prevention strategies or urban design (i.e., increased surveillance and pedestrian lighting, reduction in incivilities) – might increase local walking.

Several limitations should be considered when interpreting these study findings. Estimated associations cannot be considered causal given the cross-sectional study design. While our study advances previous cross-sectional studies by statistically adjusting for participant’s reasons for moving to their neighborhood [37,38], unmeasured factors associated with neighborhood self-selection may still exist, thus inflating estimated associations between the built environment and physical activity. Moreover, given that participants in our sample reported living in their neighborhoods for approximately 13 years (on average), memory bias and errors associated with people’s reasons for moving to their neighborhoods likely exist. The modest response rate, study selection bias, and the age of the data may limit the generalizability of our findings. Despite using reliable context-specific (neighborhood) measures of physical activity, biases associated with capturing self-reported physical activity is a limitation [39]. Despite selecting the best fitting principal component analysis solutions, the low internal consistency of some perceived walkability and neighborhood preference scales could have led to fewer associations between these scales and the walking outcomes being found in our study. The use of a simple random sample to recruit households across the Calgary metropolitan area meant that fewer high walkability neighborhoods were included in our sample, thus, resulting in fewer survey participants from this neighborhood type. The lower sample size in the high walkable neighborhoods likely reduced statistical power to find statistically significant differences or associations. For example, in some cases we found point estimates (group differences and associations) of similar magnitude to be statistically significant for low and medium walkable neighborhoods but not statistically significant for high walkable neighborhoods.

Both the perceived and objectively-determined neighborhood built characteristics appear to contribute to neighborhood-based walking – in particular walking for transportation. However, objective measures and perceptions of the built environment should not be considered interchangeable. Our findings suggest that interventions designed to change perceptions of neighborhood walkability might have a stronger influence on walking for adults in higher walkable neighborhoods. Creating walkable neighborhoods might increase participation and time spent walking for transportation locally; however, increasing local recreational walking might require other intervention approaches.

## Competing interests

The authors’ declare that there are no competing interests.

## Authors’ contributions

EJ drafted the manuscript, undertook the analysis, and interpreted results. GRM contributed to the manuscript writing, oversaw the analysis, and contributed to the interpretation of results. Both authors read and approved the final manuscript.
